# Real-World Cost-Effectiveness of Late Time Window Thrombectomy for Patients With Ischemic Stroke

**DOI:** 10.3389/fneur.2021.780894

**Published:** 2021-12-14

**Authors:** Lan Gao, Andrew Bivard, Mark Parsons, Neil J. Spratt, Christopher Levi, Kenneth Butcher, Timothy Kleinig, Bernard Yan, Qiang Dong, Xin Cheng, Min Lou, Congguo Yin, Chushuang Chen, Peng Wang, Longting Lin, Philip Choi, Ferdinand Miteff, Marj Moodie

**Affiliations:** ^1^Deakin Health Economics, Institute for Health Transformation, Deakin University, Geelong, VIC, Australia; ^2^Melbourne Brain Centre, Royal Melbourne Hospital, Parkville, VIC, Australia; ^3^Departments of Neurology, John Hunter Hospital, University of Newcastle, Callaghan, NSW, Australia; ^4^Department of Neurology, UNSW South Western Clinical School, Liverpool Hospital, University of New South Wales, Kensington, NSW, Australia; ^5^Department of Neurology, Prince of Wales Hospital, University of New South Wales, Sydney, NSW, Australia; ^6^Department of Neurology, Royal Adelaide Hospital, Adelaide, SA, Australia; ^7^Department of Neurology, Huashan Hospital, Fudan University, Shanghai, China; ^8^Department of Neurology, Second Affiliated Hospital of Zhejiang University, Hangzhou, China; ^9^Department of Neurology, Hangzhou First Hospital, Zhejiang University School of Medicine, Hangzhou, China; ^10^Zhejiang Provincial People's Hospital, Zhejiang, China; ^11^School of Medicine and Public Health, University of Newcastle, Callaghan, NSW, Australia; ^12^Department of Neurology, Box Hill Hospital, Eastern Health, Box Hill, VIC, Australia

**Keywords:** stroke, large vessel occlusions, late time window thrombectomy, real-world, cost-effectiveness

## Abstract

**Background:** To compare the cost-effectiveness of providing endovascular thrombectomy (EVT) for patients with ischemic stroke in the >4.5 h time window between patient groups who met and did not meet the perfusion imaging trial criteria.

**Methods:** A discrete event simulation (DES) model was developed to simulate the long-term outcome post EVT in patients meeting or not meeting the extended time window clinical trial perfusion imaging criteria at presentation, vs. medical treatment alone (including intravenous thrombolysis). The effectiveness of thrombectomy in patients meeting the landmark trial criteria (DEFUSE 3 and DAWN) was derived from a prospective cohort study of Australian patients who received EVT for ischemic stroke, between 2015 and 2019, in the extended time window (>4.5 h).

**Results:** Endovascular thrombectomy was shown to be a cost-effective treatment for patients satisfying the clinical trial criteria in our prospective cohort [incremental cost-effectiveness ratio (ICER) of $11,608/quality-adjusted life year (QALY) for DEFUSE 3-postive or $34,416/QALY for DAWN-positive]. However, offering EVT to patients outside of clinical trial criteria was associated with reduced benefit (−1.02 QALY for DEFUSE 3; −1.43 QALY for DAWN) and higher long-term patient costs ($8,955 for DEFUSE 3; $9,271 for DAWN), thereby making it unlikely to be cost-effective in Australia.

**Conclusions:** Treating patients not meeting the DAWN or DEFUSE 3 clinical trial criteria in the extended time window for EVT was associated with less gain in QALYs and higher cost. Caution should be exercised when considering this procedure for patients not satisfying the trial perfusion imaging criteria for EVT.

## Introduction

Seven large clinical trials have demonstrated that endovascular thrombectomy (EVT) is highly effective in increasing disability-free survival compared to the previous standard care, intravenous thrombolysis (IVT), in strokes due to a large vessel occlusion (LVO) (1–7). Based on these foundational trials, DAWN and DEFUSE 3 trials also extended the treatment time window for patients screened with perfusion imaging to identify treatment responders from 6 h out to 24 h (8, 9). These ground-breaking trials were highly selective but demonstrated considerable patient benefits. Since its introduction as routine care in Australia, providing EVT to patients with an LVO has seen a significant amount of “scope creep,” where a large proportion of patients are now offered therapy outside of the trial criteria. Previous *post-hoc* analysis has shown that providing EVT to patients meeting the trial criteria is highly cost-effective within the normal and extended time window (10–16); however, it is not known if this cost-effectiveness is maintained when patients are treated outside of the trial criteria.

Patient outcomes after stroke and EVT are highly influenced by patient characteristics (17, 18), such as age, pre-morbid disability, co-morbidities, and imaging characteristics including the site of the vessel occlusion and the volume of core/penumbra (19). It is important to acknowledge that in clinical practice, which is distinct from controlled trials that are subject to strict selection criteria, the clinicians are more likely to treat patients who do not enroll into these trials, and not all these patients benefit from the treatment to the same extent, while some are even harmed due to hemorrhage, vessel perforation, or reperfusion injury. To investigate the effect of this scope creep on the likely cost of therapy, we undertook a discrete event simulation to assess the cost-effectiveness of EVT in the real world with respect to patients meeting/not meeting the clinical trial criteria, in comparison to the medical treatment alone.

## Materials and Methods

### Study Population

Data from the International Stroke Perfusion Imaging Registry (INSPIRE) were used to source the baseline and 90-day clinical and imaging data over a 5-year period (2015–2019) (20, 21). Patients presenting with acute neurological deficit within 24 h of symptom onset underwent routine multimodal CT (non-contrast CT, perfusion CT, and CT angiography) and received thrombolysis and/or EVT if they were deemed eligible according to local clinical guidelines. Eligibility for EVT in routine clinical practice included the presence of an LVO [internal carotid artery (ICA) or M1 occlusion] that an interventionalist could potentially retrieve. Consent of the patients was obtained according to the Declaration of Helsinki. The Hunter New England Area Health Service Human Research Ethics Committee reviewed and approved the study protocol in 2012.

To assess the effect of late time window treatment, only patients presenting after 4.5 h from symptom onset were included from the INSPIRE database. In this study, the late window was defined as a patient presenting >4.5 h after stroke onset, so as to have a sufficient number of patients from INSPIRE registry to proceed with the analysis. All the other criteria from DAWN and DEFUSE 3 (as summarized in Supplementary Table I) were strictly applied to our study population. Medical treatment included non-EVT treatment with or without intravenous thrombolysis. Patients were split into groups of those who received and did not receive EVT. Patients were then matched (i.e., matching nearest neighbors) between EVT and non-EVT groups, based on age, sex, baseline National Institute of Health Stroke Scale (NIHSS), and computer tomography perfusion (CTP) ischemic core volume using the “psmatch2” command from Stata (StataCorp. 2019. Stata Statistical Software: Release 16. College Station, TX: StataCorp LLC). Premorbid modified ranking score (mRS) was insufficiently recorded in the INSPIRE, thus preventing it from being adopted as a matching variable. Next, all the patients that met and did not meet the DAWN and DEFUSE 3 criteria were, respectively, matched separately based on EVT and non-EVT following the same Stata command. Non-EVT patients with corresponding perfusion imaging criteria positive were employed as the comparator for EVT patients with positive selection criteria while those being criteria negative were adopted as a comparator for EVT patients being perfusion imaging criteria negative. Perfusion imaging selection criteria were as per the original DEFUSE 3 and DAWN trials and are summarized in Supplementary Table I. Distributions of propensity score were assessed after matching with EVT status and perfusion imaging selection criteria. The sociodemographic and clinical characteristics of the simulated cohort were defined by participants from the INSPIRE (summarized in Table 1).

**Table 1:** Baseline characteristics of the cohort.

	**Thrombectomy procedure (*****N** **=*** **131)**		**Medical treatment (*****N** **=*** **160)**		**Thrombectomy procedure (*****N** **=*** **111)**		**Medical treatment (*****N** **=*** **94)**	
	**DEFUSE 3_pos (*N =* 105)**	**DEFUSE 3_neg (*N =* 26)**	**DEFUSE 3_pos (*N =* 98)**	**DEFUSE 3_neg (*N =* 62)**	**DAWN_pos (*N =* 91)**	**DAWN_neg (*N =* 20)**	**DAWN_pos (*N =* 59)**	**DAWN_neg (*N =* 35)**
Age (years, mean)	68.0 (15.27)	70.0 (14.16)	69.9 (13.40)	68.0 (13.62)	65.1 (15.17)	67.3 (17.41)	69.0 (13.99)	69.1 (14.79)
Gender (male, %)	38 (36.2%)	8 (30.8%)	43 (43.9%)	18 (29.0%)	39 (42.9%)	5 (25.0%)	24 (40.7%)	13 (37.1%)
Baseline NIHSS	16 (11–21)	16 (11–21)	15 (11–19)	14 (8–19)	17 (14–21)	18 (14–22)	17 (13–20)	18 (8–22)
Baseline core volume (ml, median)	19 (8–36)	91 (73–126)	21 (11–46)	15 (0.1–76)	21 (10–36)	92 (82–128)	23 (10–40)	76 (19–95)
Perfusion lesion volume (ml, median)	112 (76–149)	199 (162–229)	113 (81–160)	24 (7–151)	123 (86–150)	207 (191–234)	113 (75–137)	137 (104–196)
Penumbra volume (ml, median)	90 (62–117)	93 (63–123)	83 (51–124)	11 (3–73)	98 (62–120)	99 (88–125)	79 (58–113)	86 (44–109)
**Treatment type (** ***n*** **, %)**
Both EVT and tPA	50 (47.6%)	16 (61.5%)	0	0	43 (47.2%)	12 (60.0%)	0	0
EVT only	55 (52.4%)	10 (38.5%)	0	0	48 (52.8%)	8 (40.0%)	0	0
tPA only	0	0	7 (7.1%)	5 (8.1%)	0	0	4 (6.8%)	3 (8.6%)
Symptom onset to CTP (mins, mean)	322 (86)	394 (72)	403 (79)	372 (83)	335 (94)	387 (92)	384 (73)	393 (81)
Target mismatch (n, %)	105 (100%)	0	98 (100%)	3 (4.8%)	86 (94.5%)	1 (5%)	56 (94.9%)	10 (28.6%)
Core volume>70ml (n, %)	0 (0)	20 (76.9%)	0 (0)	24 (38.7%)	0 (0)	19 (95.0%)	0 (0)	24 (68.6%)
Proportion receiving EVT 4.5–6 hours (n, %)	31 (29.5%)	9 (34.6%)	–	–	23 (25.3%)	6 (30.0%)	–	–
**Occlusion location (** ***n*** **, %)**
ICA	35 (33.3%)	15 (57.7%)	28 (28.6%)	27 (43.6%)	37 (40.7%)	12 (60.0%)	24 (40.7%)	21 (60.0%)
M1	70 (66.7%)	11 (42.3%)	70 (71.4%)	35 (56.5%)	54 (59.3%)	8 (40.0%)	35 (59.3%)	14 (40.0%)
**3 m mRS**
0	14 (13.33%)	2 (7.69%)	11 (11.22%)	12 (19.35%)	15 (16.48%)	2 (10.0%)	9 (15.25%)	4 (11.43%)
1	20 (19.05%)	3 (11.54%)	15 (15.31%)	13 (20.97%)	12 (13.19%)	1 (5.0%)	7 (11.86%)	4 (11.43%)
2	14 (13.33%)	3 (11.54%)	10 (10.2%)	2 (3.23%)	9 (9.89%)	2 (10.0%)	6 (10.17%)	1 (2.86%)
3	30 (28.57%)	5 (19.23%)	13 (13.27%)	9 (14.52%)	20 (21.98%)	4 (20.0%)	9 (15.25%)	3 (8.57%)
4	12 (11.43%)	3 (11.54%)	27 (27.55%)	8 (12.9%)	11 (12.09%)	3 (15.0%)	12 (20.34%)	4 (11.43%)
5	5 (4.76%)	1 (3.85%)	8 (8.16%)	7 (11.29%)	6 (6.59%)	0 (0%)	4 (6.78%)	9 (25.71%)
6	10 (9.52%)	9 (34.62%)	14 (14.29%)	11 (17.74%)	18 (19.78%)	8 (40.0%)	12 (20.34%)	10 (28.57%)

*DEFUSE_neg/pos, patient not meeting/meeting the DEFUSE 3criteria; DAWN_neg/pos, patien not meeting/meeting the DAWN criteria; EVT, endovascular clot retrieval; tPA, tissue plasminogen activator; CTP, computer tomography perfusion; mRS, modified Rankin scale*.

*Unlike normal matching (in which single characteristics that distinguish two groups are matched, e.g., baseline infarct core volume), propensity score matching attempts to reduce the potential bias due to a range of confounding variables (e.g., age, sex, baseline NIHSS and baseline ischemic core volume). Subsequently, the comparability of one particular variable could be suboptimal than that from the normal matching approach*.

*The number of patients meeting the DEFUSE 3 or DAWN criterion in the thrombectomy/medical treatment groups overlaps*.

### Model Structure

The discrete event simulation (DES) model was initiated from Day 90 in one of seven health states as defined by the mRS score (0–6) on the basis of the INSPIRE registry data (Table 1) (22). The DES model was selected to avoid the use of fixed cycle lengths and improve the calculation efficiency. Both DES and Markov models produce results that are highly consistent and cost-effective and support the same resource allocation decisions (23–27). The resource constraints were not considered in the current DES model (e.g., EVT is always available to patients in need). In the long-term, myocardial infarction (MI) and recurrent stroke were simulated given the substantially increased risk of coronary heart disease (CHD) post stroke (i.e., the 5-year risk of MI or vascular death was 17.4%) (28). Following each event, the patient could die or survive from such events, or die from other non-cardiovascular disease (CVD) causes. TreeAge Pro was used for discrete event simulation (TreeAge Pro 2019, R2. TreeAge Software, USA). The model structure is presented in Supplementary Figure I. Detailed model description is provided in the Supplementary Material.

### Model Inputs

#### Time-to-Event Distribution and Transition Probability

Given the 3-month follow-up of INSPIRE, the long-term event rates were sourced from published literature. Time to recurrent stroke was constructed using an exponential distribution (29), while a Gompertz distribution following a previously published study was tested in the sensitivity analysis (30). Time to MI was constructed using an exponential distribution derived from a registry that prospectively recorded incidence of MI post stroke (*N* = 9,840) during the period of 2003–2016 (median follow-up: 4.7 years) (31).

For patients within or outside of clinical trial criteria for EVT and controls, the identical time-to-event distribution for all the possible events was applied, and this was considered conservative as there was insufficient evidence to support the carry-on effect of EVT. Parameters for the time-to-event distributions are provided in Supplementary Table II. Mortality rates (due to non-CVD and CVD causes) by age are summarized in Supplementary Tables III, IV.

#### Costs

The healthcare costs related to the index stroke (including the cost for EVT procedure), rehospitalization due to MI and stroke, and long-term management costs (including medications and GP/specialist consultations) were considered in the model-based simulation study (32, 33). For recurrent stroke, the costs of acute care (i.e., hospitalization) based on the severity of stroke (as defined by the mRS score) were extracted from national administrative databases (34). The long-term management costs for stroke (according to the functional status defined by mRS score) and MI were sourced from the published literature (32–34). All the costs (valued in Australian dollars in 2018, where 1 AUD = 0.75 USD was the average exchange rate in 2018) applied in the model are presented in Supplementary Table II.

#### Utility Weights

Utility weights are preference weights representing the strength of desirability toward different health states (i.e., more preferred health states will have greater weight). They are measured on a cardinal scale of 0–1, where 0 indicates death and 1 indicates perfect health (negative values represent a health state worse than death) (14). In the current study, utility weights associated with being in the post-stroke health states by mRS score were informed based on published literature. A utility decrement was applied immediately following a CVD event to account for the temporarily reduced quality of life after an event (35, 36). The utility weights are shown in Supplementary Table II.

#### Cost-Effectiveness Analysis

In the base case, 50,000 Australian patients with suspected LVO were modeled in the DES. The perspective of the Australian healthcare system was considered to measure the costs and benefits over a 25-year time horizon. Utility weights were utilized to estimate the quality-adjusted life years (QALYs) (14). In addition, the life years lived were estimated to measure the survival gains. The primary outcome for the cost-effectiveness analysis was the incremental cost-effectiveness ratio (ICER) per QALY gained, which is calculated as the ratio between incremental cost and incremental QALYs gained (intervention vs. control). Separate ICERs were estimated for patients undergoing EVT by satisfying the DAWN and DEFUSE 3 criteria. Costs and benefits were discounted at a rate of 3% per annum (37). The often quoted willingness-to-pay (WTP) per QALY threshold of AUD50,000 was adopted to assess the cost-effectiveness of EVT measured against medical treatment without EVT (38).

### Sensitivity Analyses

One-way deterministic sensitivity analyses (i.e., varying one model parameter at a time within a plausible range) were undertaken to examine the robustness of base case results. The results of deterministic sensitivity analyses were presented in the form of a tornado diagram. In addition, probabilistic sensitivity analyses by constructing the distribution for the key uncertain parameters were run to further explore the results. A key assumption made in the probabilistic sensitivity analyses was that the distributions for each parameter were not correlated (i.e., the variation in one parameter is not associated with a change in another parameter). An incremental cost-effective plane and a cost-effectiveness acceptability curve were generated to illustrate the results of probabilistic sensitivity analyses.

### Model Validation

The long-term data for patients post stroke were utilized to validate the outcomes from the simulation study. In particular, studies reporting the long-term survival and recurrence of stroke and MI were retrieved from a rapid literature review through key databases to examine the model outputs.

Expanded methods are shown in the Supplementary Materials.

### Data Availability

INSPIRE data may be available following a reasonable request to the corresponding author in the anonymized form by any qualified investigator.

## Results

### Study Population

In this study, a total of 372 patients were included from the INSPIRE registry. Of the 372 patients, 291 patients were positive for DEFUSE 3 and 205 were also positive for DAWN criteria. There were 124 patients (33.3% of the total study population) who were positive for both DEFUSE 3 and DAWN criteria (and were within 6- and 16-h time windows); 85 of this received EVT while 39 did not receive EVT.

**Figure 1:**
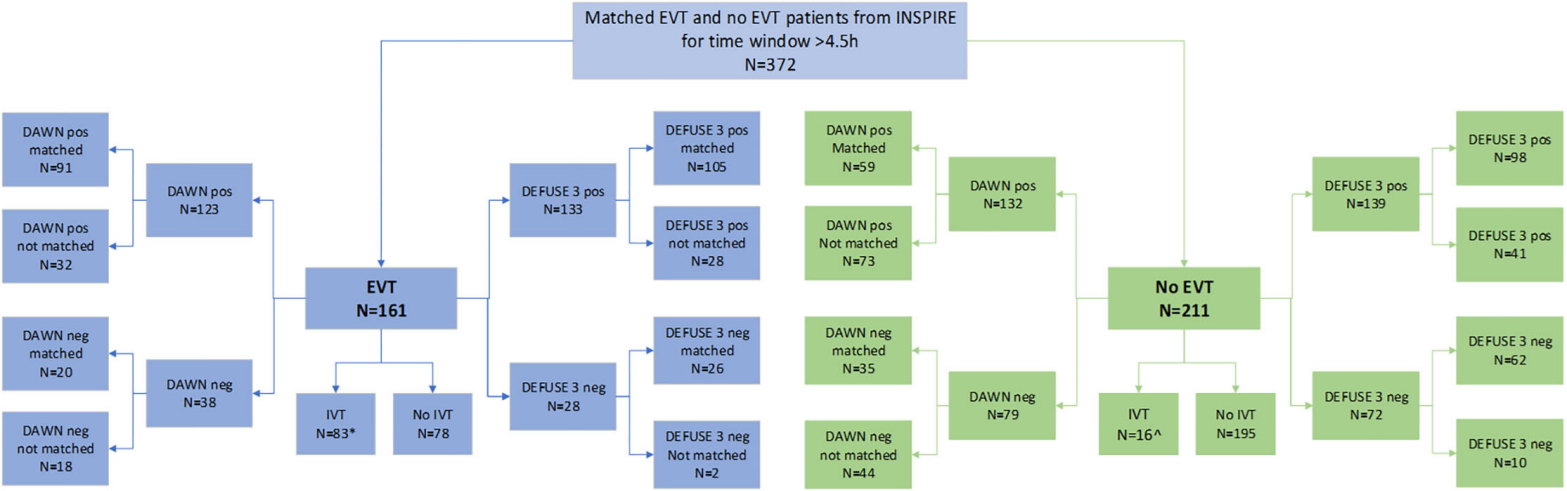
Illustration of propensity score matching by treatment and perfusion criteria.

Of the 372 patients, 161 were treated with EVT beyond 4.5 h. Among the EVT patients, 83 were also treated with thrombolysis, of which 11 were treated beyond 4.5 h. Of the 372 patients, 211 were not treated with EVT and presented to the hospital beyond 4.5 h. Of these, 16 (7.6%) were treated with thrombolysis beyond 4.5 h. Next, of the 161 patients in the EVT cohort, 133 met the DEFUSE 3 criteria (105 were matched and 28 were unmatched for the analysis) while 28 did not (26 were matched and 2 were unmatched). Among these 161 patients, 123 met the DAWN criteria (91 were matched and 32 were unmatched) while 38 did not (20 were matched and 18 were unmatched). Patients who were not matched for DAWN or DEFUSE 3 were not included in the long-term cost-effectiveness analysis by that criteria. The process of propensity matching is illustrated in Figure 1.

The patient details and cohort sizes are described in Table 1. The characteristics of patients unmatched based on EVT status and perfusion criteria are shown in Supplementary Table V. The distribution of propensity score after matching based on EVT and DAWN/DEFUSE 3 criteria is shown in Supplementary Figure II.

Additional results of population characteristics are supplied in the Supplementary Materials.

### Cost-Effectiveness Analysis

Of the patients treated with EVT, the presence or absence of DEFUSE 3 mismatch was associated with different outcomes: $70,810 in costs and 8.81 QALYs for DEFUSE-positive patients, $59,302 and 6.03 QALY for DEFUSE-negative patients. Comparatively, the cost of best medical practice with/without thrombolysis was $50,347 and 7.05 QALYs for DEFUSE-positive and $48,767 and 7.61 QALY for DEFUSE-negative patients, respectively (Table 2). Therefore, compared with patients who did not undergo EVT regardless of their trial eligibility, offering EVT to patients satisfying the DEFUSE 3 criteria was cost-effective (ICER $11,608 vs. DEFUSE 3 positive without EVT or $18,303/QALY vs. DEFUSE 3 negative without EVT). However, using the same set of comparators, it was not cost-effective (more costly and less effective) to treat patients with EVT who did not meet the DEFUSE 3 criteria in the extended time window (Table 2).

**Table 2:** Base case results from the cost–effectiveness analysis.

	**Thrombectomy procedure**		**Medical treatment**		**Thrombectomy procedure**		**Medical treatment**	
	**DEFUSE 3 positive**	**DEFUSE 3 negative**	**DEFUSE 3 positive**	**DEFUSE 3 negative**	**DAWN positive**	**DAWN negative**	**DAWN positive**	**DAWN negative**
Total QALYs	8.81	6.03	7.05	7.61	7.56	5.59	7.02	4.64
Total LYs	12.77	9.19	12.09	11.60	11.30	8.45	11.22	10.05
Total costs	$70,810	$59,302	$50,347	$48,767	$66,096	$56,788	$47,517	$43,801
Average number of events^*^
Deaths	0.626	0.730	0.645	0.660	0.669	0.752	0.671	0.705
MI	0.216	0.153	0.202	0.194	0.190	0.141	0.188	0.168
Stroke	0.822	0.594	0.779	0.747	0.728	0.544	0.723	0.649
Cost of hospitalization	$36,729	$34,622	$18,015	$17,720	$35,864	$34,161	$17,509	$16,826
Cost of management	$24,082	$24,680	$32,332	$31,047	$30,232	$22,627	$30,008	$26,974
**ICER**
	$11,608	dominated^∧^	–	–	$34,416	dominated^∧^	–	–

*QALY, quality–adjusted life year; LY, life year; ICER, incremental cost–effectiveness ratio*.

* *this is the average number of event per patient (not all patients experienced the CVD event)*.

∧ *the dominance was based on higher number of death occurred even though the ICER was $13,588*.

*Dominated refers to the results showing the intervention is more expensive whereas has less benefits than the comparator*.

The results according to DAWN criteria showed a similar trend: $66,096 and 7.56 QALYs for DAWN-positive patients, $56,788 and 5.59 QALY for DAWN-negative patients, in comparison to $47,517 and 7.02 QALYs in those who did not receive EVT but being DAWN-positive and $43,801 and 4.64 QALYs in those being DAWN-negative (Table 2). In summary, compared with the patients who did not undergo EVT, EVT was a cost-effective treatment for patients meeting the DAWN perfusion criterion (ICER $34,416 vs. DAWN positive without EVT or $7,611/QALY vs. DAWN negative without EVT). However, again, it was not cost-effective to treat patients with EVT when they did not fulfill the DAWN criteria. It was more costly and less effective when compared to DAWN-positive patients who did not undergo EVT. It was also more costly and incurred higher rates of death (detailed below) when compared to DAWN-negative patients who did not undergo EVT, even with an ICER of $13,588/QALY below the WTP/QALY threshold.

The results of cost-effectiveness analysis by 3-month mRS score from INSPIRE are summarized in Supplementary Tables VI, VII. Generally, patients who achieved better functional outcomes at 3-month follow-up incurred lower annual costs post the index stroke.

Over the modeled time horizon, more simulated deaths occurred among patients who received EVT but were outside of either of the criteria [*N* = 7,300 (DEFUSE 3-negative) or *N* = 7,520 (DAWN-negative) per 10,000 patients], compared to those treated without EVT [*N* = 6,450 (DEFUSE-positive) or *N* = 6,600 (DEFUSE-negative), *N* = 6,710 (DAWN-positive) or *N* = 7,050 (DAWN-negative) per 10,000 patients] (Table 2).

### Sensitivity Analysis

In comparison with medical treatment, the cost-effectiveness of extended time window EVT in patients not meeting the DAWN or DEFUSE 3 criteria was very sensitive to the time horizon, discount rate, probability of recurrent stroke being fatal, and cost of acute care for recurrent stroke (Supplementary Figures III, IV).

Compared to the medical treatment without EVT, probabilistic sensitivity analysis consistently showed that EVT was highly likely to be cost-effective in patients satisfying the clinical trial criteria for EVT, with a corresponding probability of 100% for DEFUSE 3 and DAWN criteria, respectively. On the other hand, it consistently showed that the patients receiving EVT, but not meeting the trial criteria, achieved inferior health outcomes in the long-term, rendering EVT not cost-effective (with 0% probability of being cost-effective for both criteria) when compared with medical treatment (Supplementary Figures V, VI).

Results of using a 5-year time horizon and Gompertz distribution for the probability of recurrent stroke are provided in Supplementary Tables VIII, IX.

### Model Validation

The 5-year survival predicted by the current model was around 51.8% for patients who received non-EVT treatment. In comparison, the Oxford Vascular Study reported a 5-year survival rate of over 50% (39), and a Swedish study found a 49.4% survival for ischemic stroke (40). Moreover, the predicted 5-year QALY gains from the non-EVT group were 2.07 in our study vs. 2.21 from the long-term observation (39).

In addition, our model also predicted the number of recurrent strokes and MI, which are highly comparable with the systematic review of long-term observational studies (29, 31, 41).

Additional results of model validation are provided in the Supplementary Materials.

## Discussion

In this study, we found that treating patients with EVT who did fulfill the DAWN and DEFUSE 3 trial criteria had significantly better outcomes compared to non-EVT patients, which was also highly cost-effective. However, for patients who did not fulfill the trial criteria, EVT was unlikely to be cost-effective based on the primary comparator (medical therapy patients being perfusion criteria positive), given the higher costs and number of deaths, and lower QALY gains based on the widely accepted threshold of $50,000/QALY. Importantly, this study reflects the practice in the real world and is not within a tightly controlled clinical trial, and so represents the implementation of the evidence rather than the evidence itself. The patients in the trial negative cohort were predominantly with a large established ischemic core. Important to note is that while the trial negative groups have been shown to have a reduced rate of cost-effective clinical benefit from EVT, there may be individual patients within these groups who do benefit, and this requires further investigation. Even though there are currently randomised controlled trials (RCTs) underway to ascertain the benefit of EVT in the large core at baseline (i.e., the SELECT 2; RESCUE Japan-LIMIT NCT03702413; TENSION NCT03094715) (42), the results from the current study including, largely, patients with baseline infarct core over 70 ml of imaging selection criteria negative could stress the importance of careful patient selection in offering EVT in real-world.

Evaluations of the cost-effectiveness of EVT were based on a decision tree combined with a Markov model, drawing efficacy data from clinical trials (i.e., 100% of the patients fulfilled the EVT criterion) and localized costs (10–13, 16, 32, 33, 36, 43–47). Most of the economic evaluations concluded that EVT was a dominant treatment option (i.e., higher QALYs and lower costs) for patients with ischemic stroke, while the rest reported its positive cost-effectiveness (i.e., ICER falls below the WTP/QALY threshold for various jurisdictions), including late window EVT (studies listed in Supplementary Table X). The gains in QALYs from these studies ranged from 0.54 (43) to 2.51 (46) over the lifetime horizon. However, positive cost-effectiveness outcomes that are built upon efficacy outcomes from clinical trials may misinform clinical and policy decision-making, thereby leading to inappropriate management of patients with ischemic stroke. This in turn may result in unnecessary use of health resources (high medical costs of EVT) and worsening health outcomes, representing significant inefficient allocation of resources.

Our data suggest that offering EVT based on the DEFUSE 3 criteria is associated with greater QALY gains than that by the DAWN criteria as a confirmatory result from a non-selected registry of routine care. In contrast, treating patients outside of the DAWN criteria leads to poorer outcomes in terms of survival gains. The difference is driven by the post-stroke outcome after 3 months, where more patients achieved functional independence (i.e., mRS ≤ 2). In terms of 3-month functional outcomes, there was no significant difference between the DAWN-positive and DAWN-negative patients (ordinal logit regression, *p* = 0.055). The long-term modeling enabled the translation from short-term temporary health status to the eventual health outcomes. The modeling suggested that neurologists/neuro-interventionists should exercise more caution when offering EVT to patients not satisfying the perfusion criteria.

It is important to highlight that EVT treatment for patients in the standard time window of up to 6 h of symptom onset is widely thought to be cost-effective no matter what the perfusion imaging characteristics are. However, in the early time window, there is still limited health economic assessment on a real-world dataset of comparable depth to that seen in the current extended time window (i.e., with CTP data available). The primary source of such patient outcome variation includes the differences in salvageable ischemic lesion volume identified by CTP (18, 48, 49).

The favorable cost-effectiveness of offering EVT to DAWN-negative patients compared to those receiving medical treatment being DAWN-negative should be interpreted with caution. It is worth noting that even though the resultant ICER in this scenario was cost-effective, treatment with EVT in this cohort led to a greater number of deaths (*N* = 470 per 10,000 patients treated) over the simulated time horizon, which rendered it inferior to medical treatment. The analysis based on all patients without EVT procedure regardless of DAWN criterion revealed consistent results as the analysis according to the primary comparator—offering EVT to DAWN-negative patients has zero probability of being cost-effective.

There are limitations to this study. First, the severity of recurrent stroke was not modeled explicitly but was accounted for when assigning the costs and utility weights implicitly (i.e., when a recurrent stroke occurred, the hospitalization and management costs and utility weights were determined by the severity of that stroke). Second, the effectiveness of EVT was based on prospectively collected cohort data; there might be some concerns regarding the comparability of the compared cohorts (e.g., EVT patients with perfusion criteria negatives had higher baseline core volume than that for the primary comparator). The non-significant between-group difference in other baseline characteristics may partly ease this concern. Third, INSPIRE recruited more non-EVT patients with mild stroke compared to the landmark RCTs, which may confound the comparison. However, participants were matched in terms of onset age, gender, baseline NIHSS, and infarct core volume with propensity score matching approach. Fourth, the time-to-event distributions for recurrent stroke and MI were sourced from non-Australian-based studies, but between-country differences are likely to be minimal given the similar socio-economic settings. Fifth, the subgroup cost-effectiveness analysis pertaining to the variations in onset time and age, NIHSS score, and clinical infarct mismatch ratio are not performed due to limited sample size (e.g., the smallest sample size in the EVT group of DAWN negative patients was 20). Last, the recurrence of stroke was slightly overestimated in the DES model; however, in the sensitivity analysis, lowering the probability of recurrent stroke did not alter the conclusion about the cost-effectiveness of EVT in patients outside of the clinical trial criteria.

## Conclusions

Treating patients meeting the clinical trial perfusion imaging criteria in the extended time window with EVT is highly cost effective, while patients not meeting these criteria may not be cost effective, thereby highlighting the importance of the selection of patients. It is recommended that careful selection should be exercised when considering this procedure for patients not satisfying the perfusion imaging criteria for extended time window EVT. The real-world data analysis also confirmed that EVT is cost-effective for patients fulfilling the DEFUSE 3 or DAWN criteria in Australia.

## Data Availability Statement

The raw data supporting the conclusions of this study can be requested from Associate Professor Andrew Bivard (abivard@unimelb.edu.au) by a qualified researcher.

## Ethics Statement

The studies involving human participants were reviewed and approved by the Hunter New England Area Health Service Human Research Ethics Committee. The patients/participants provided their written informed consent to participate in this study.

## Author Contributions

LG, AB, MP, and MM conceived and designed the study. LG conducted data analysis and drafted the manuscript. AB, MP, NJS, CL, KB, TK, BY, QD, XC, ML, CY, CC, PW, LL, PC, and FM contributed to the data collection and provided important inputs to the manuscript. All authors provided consent to submit the paper.

## Funding

NJS was supported by a co-funded Australian NHMRC/NHF Career Development/Future Leader Fellowship GNT1110629/100827.

## Conflict of Interest

The authors declare that the research was conducted in the absence of any commercial or financial relationships that could be construed as a potential conflict of interest.

## Publisher's Note

All claims expressed in this article are solely those of the authors and do not necessarily represent those of their affiliated organizations, or those of the publisher, the editors and the reviewers. Any product that may be evaluated in this article, or claim that may be made by its manufacturer, is not guaranteed or endorsed by the publisher.
