# Supplementary material for: Real-World Cost-Effectiveness of Late Time Window Thrombectomy for Patients With Ischemic Stroke
Source: Front Neurol. 2021 Dec 14;12:780894. doi: 10.3389/fneur.2021.780894 (PMC8712752; doi:10.3389/fneur.2021.780894)
Supplement: Supplementary file 1 [file Data_Sheet_1.PDF]

SUPPLEMENTAL MATERIAL

Expanded Methods & Results

Expanded methods

Model structure

Recurrent stroke and MI were simulated in the long-term modelling. Post-stroke management costs including outpatient care (GP visit, specialist consultation, examinations, medications) and nursing home care were assigned according the 3-month mRS score. In the case of an event (recurrent stroke or MI), the corresponding hospitalisation cost related to that event was incurred. Over the entire simulation, one patient can experience more than one recurrent stroke and/or MI. The post-event management cost was adjusted if the stroke severity increased or an MI was occurred (e.g. if patients experienced an MI, the management cost for MI was added to the cost of managing stroke alone). Meanwhile, a one-off disutility associated with a recurrent stroke or MI was applied when such an event was simulated in the model to reflect the decrease in the quality of life during an acute cardiovascular event. Both management costs and utilities were accrued according to the length of time stayed at a given health state.

In addition, for each simulated patient, a record was created to register the number of recurrent stroke and MI experienced, the time of the event, and finally the time of death if the death occurred over the time horizon. The survival over the first 5-year time horizon was estimated as the total number of deaths divided by the total number of simulated patients (i.e. 50,000) to validate against the survival observed in the published literature.

Model inputs

Time-to-event distribution and transition probability

The time-to-event for recurrent stroke, MI, and death was randomly sampled from the constructed distribution for the respective events. The event with the shortest time to occur would ensue next. For example, if the sampled distributions were 10 years, 5 years and 20 years for recurrent stroke, MI, and death separately. The first event a patient would experience is MI. After the year 5, the model would sample from the former two time-to-events distributions again to determine the next event to simulate following the same rationale whereas the time to death was sampled once for one patient and remained the same throughout the entire time horizon. For each hypothetical patient, the simulation stopped when the death occurred or when the end of the time horizon reached.

The probability of dying from the corresponding MI or stroke were sourced from the published literature. Background mortality data were obtained from the Australian Bureau of Statistics (cause of death) and constructed as distributions using the tableProb function in TreeAge –the

time to death is dependent on the starting age in the model (Supplementary Tables VII and VIII).

Results

Population characteristics

Of the 211 patients not receiving EVT, 139 fulfilled the DEFUSE 3 criteria (98 were matched and 41 were unmatched), and 72 did not (62 were matched and 10 were unmatched). In the same cohort 132 met the DAWN criteria (59 were matched and 73 were unmatched) and 79 did not (35 were matched and 44 were unmatched). In addition, 81 patients who were not matched for DEFUSE 3 or 167 patients who were not matched for DAWN respectively were not included in the long-term cost-effectiveness analysis by that criteria.

Among the EVT patients not meeting the DEFUSE 3 criteria but matched for analysis, 20 (77%) were due to a large ischemic core. The remaining 6 EVT patients treated did not have a large ischemic core or mismatch tissue present. From the DAWN negative patients, 19 (95%) were due to a large ischemic core and 1 (5%) was due to presence of clinical-core mismatch.

Model validation

A systematic review and meta-analysis reporting the results of MI after stroke or transient ischemic attack from 39 studies involving 65,996 patients, reported the annual risk of total MI as 2.2% (95% confidence interval 1.7 to 2.7) with the raw range of individual studies from 0.5% to 4.7% [1]. Another UK study suggested a cumulative MI incidence of 5.4% over 10 years[2]. For recurrent stroke, a systematic review showed pooled cumulative recurrent risk of 26.4% (95%CI 20.1 to 32.8) at 5 years, and 39.2% (95%CI 27.2-51.2%) at 10 years post first-ever stroke. The 5-year and 10-year results from the DES model found that, for example in the medical treatment group with DEFUSE 3 criterion positive, 5.92% and 26.94% of simulated patients respectively had MI and recurrent stroke at 5-years, while 10.82% and 47.21% of the simulated population experienced MI and recurrent stroke at 10-years following the initial stroke. It is considered that the prediction from the DES model was well within the range of long-term observational studies in stroke patients (with a slight overestimation of recurrent stroke at 10-year).

Supplementary Figure I Structure of the discrete event simulation model

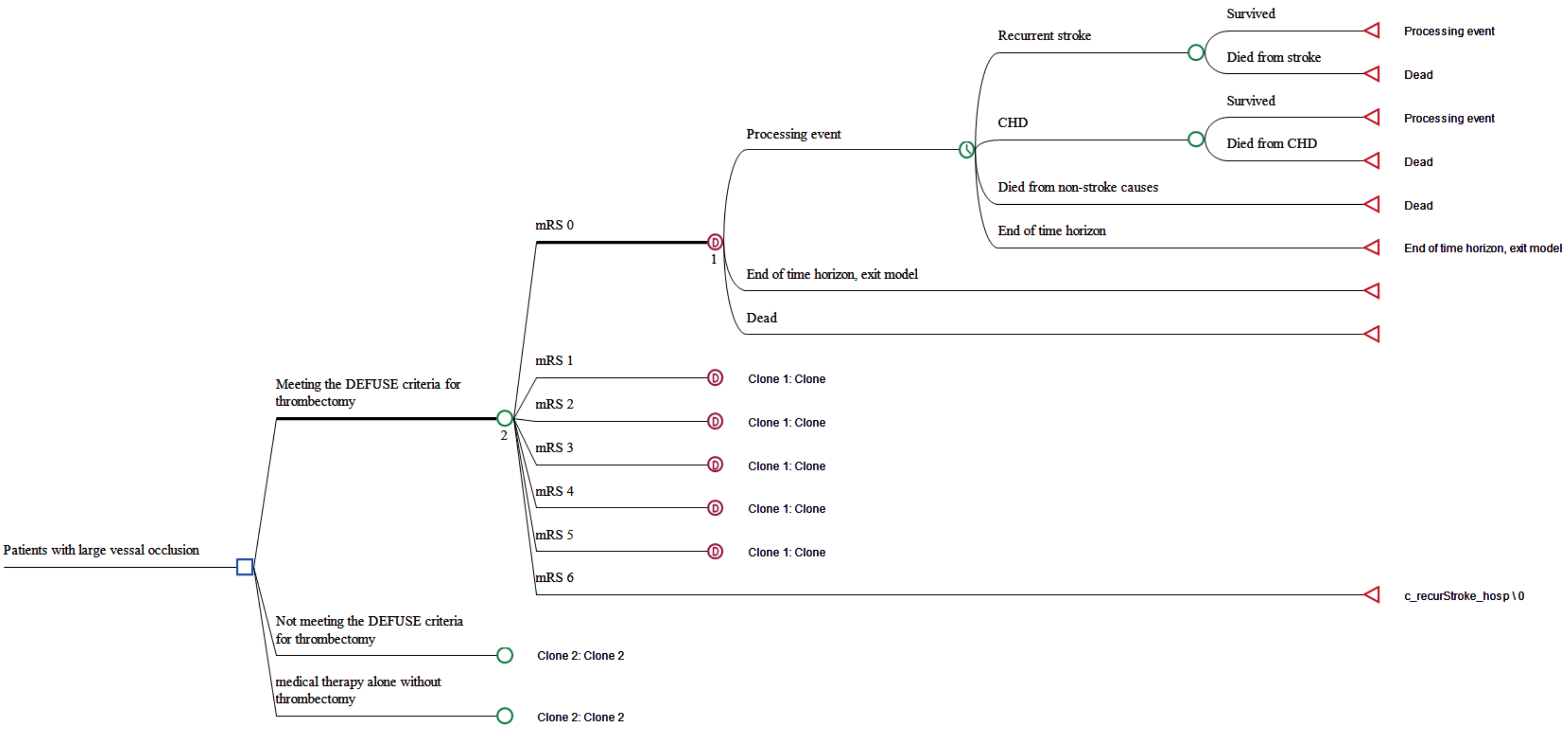

Note: hypothetical patients are distributed across seven mRS score defined health stated at the beginning according to the 3-month functional outcome. For patients died at 3-month post the index stroke, only the cost related to the initial acute hospitalisation was included with no

survival gain. For each of the mRS score defined state, patients commenced from the ‘Professing event’ branch. Three defined time-to-event distributions were randomly sample to draw the time to the next event (i.e. recurrent stroke, myocardial infarction, or death). The event with the shortest time interval will ensue firstly. For example, if the time to a recurrent stroke has the shortest time to occur, that patient will process through the recurrent stroke branch and face the risk of dying immediately following a stroke.

Supplementary Figure II. Distribution of propensity score after matching by EVT and perfusion selection criteria status

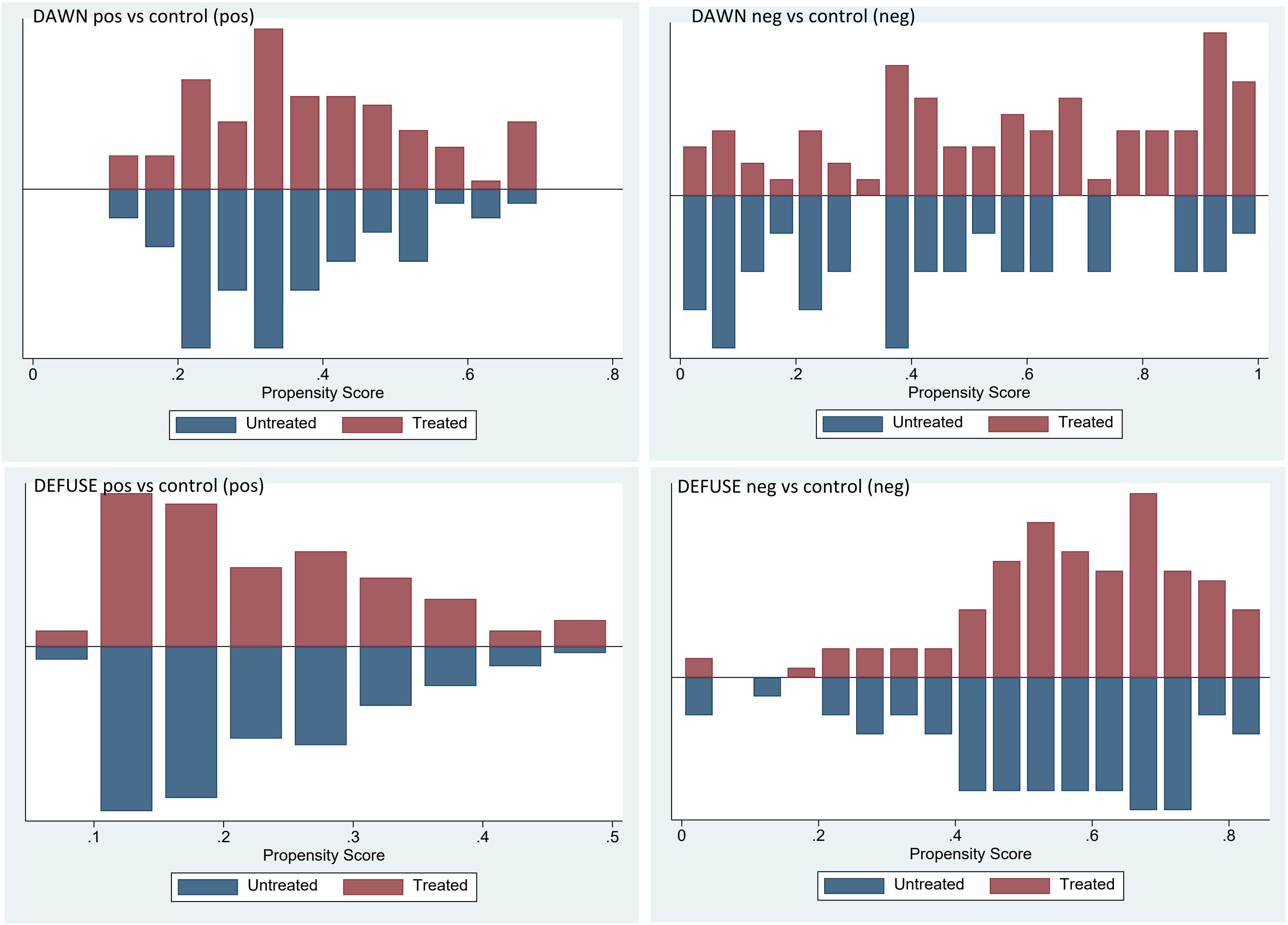

Supplementary Figure III Tornado diagram for the one-way sensitivity analysis_DEFUSE 3_negative versus medical treatment

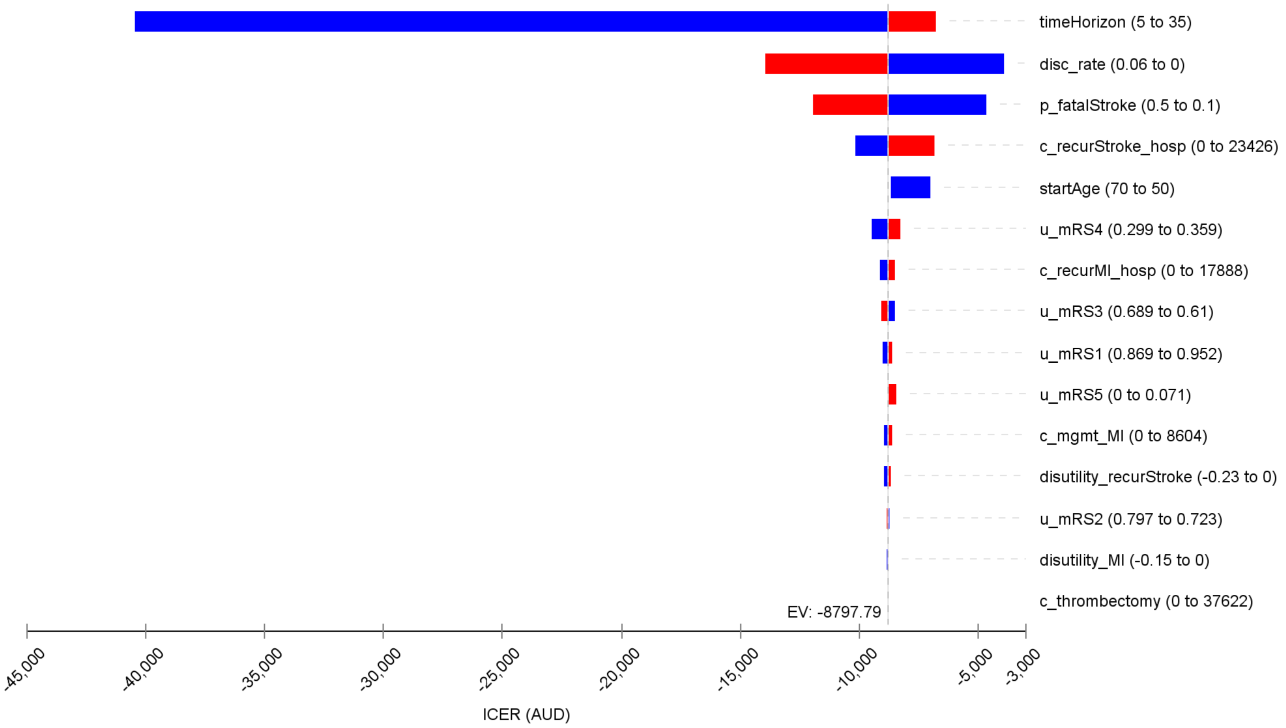

Note: blue bar indicates the variable value decreases from baseline and red bar indicates the variable value increases from baseline. Negative ICER means the higher costs (i.e. positive nominator) and lower benefit (i.e. negative denominator).

Supplementary Figure IV Tornado diagram for the one-way sensitivity analysis_DAWN_negative versus medical treatment

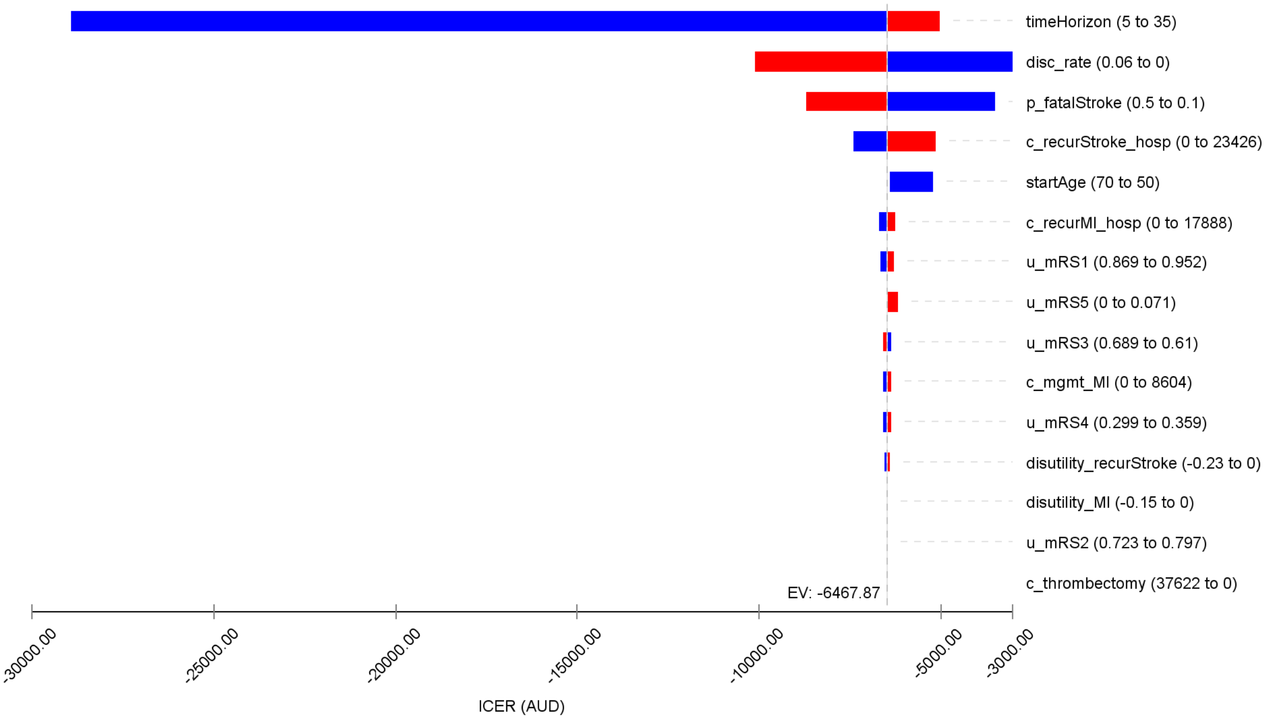

Note: blue bar indicates the variable value decreases from baseline and red bar indicates the variable value increases from baseline. Negative ICER means the higher costs (i.e. positive nominator) and lower benefit (i.e. negative denominator).

Supplementary Figure V. Incremental cost-effectiveness plane for the comparison between thrombectomy and medical therapy in people outside of DAWN criteria

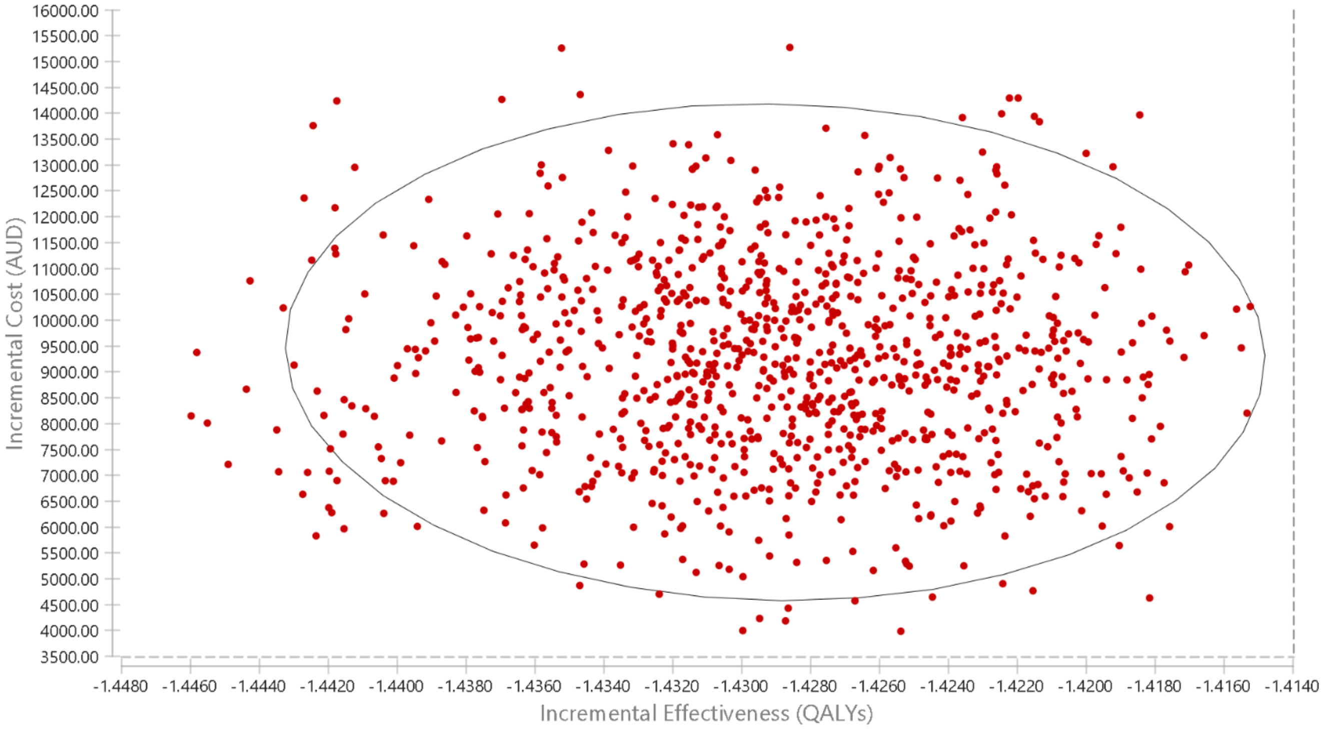

Supplementary Figure VI. Incremental cost-effectiveness plane for the comparison between thrombectomy and medical therapy in people outside of DEFUSE 3 criteria

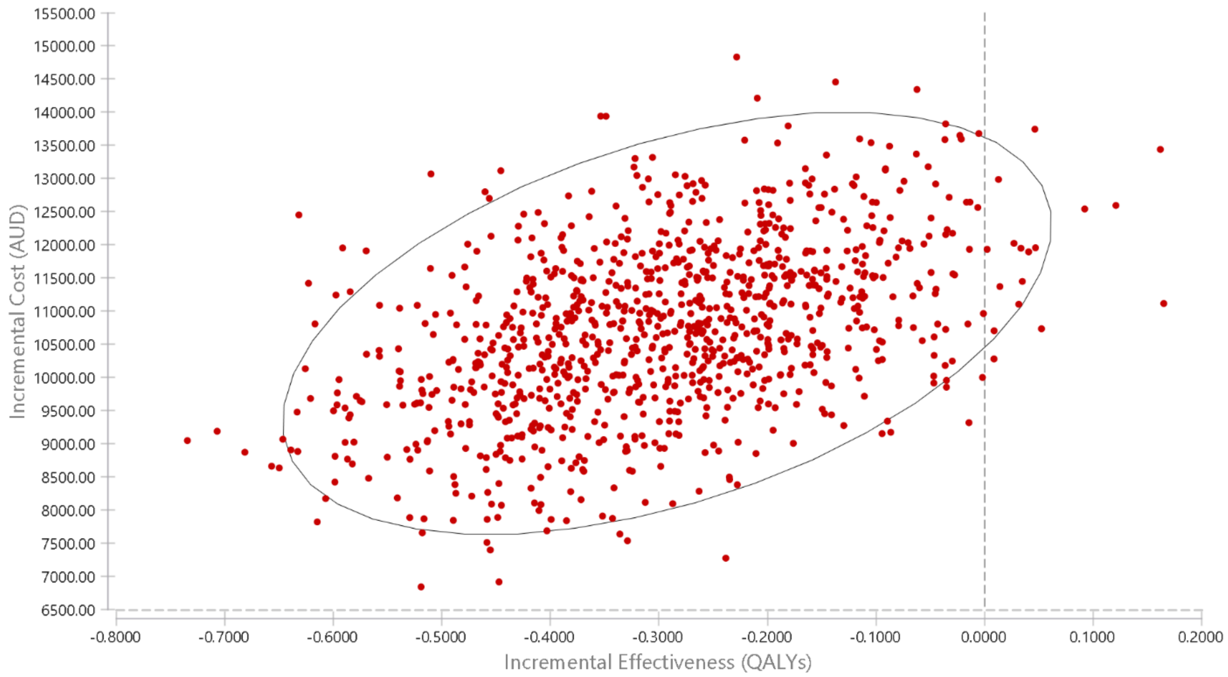

Supplementary Table I. Comparison between DAWN versus DEFUSE 3criteria

**DAWN**	**DEFUSE**
Age>80, NIHSS>10, infarct volume <21 ml; **or**	Initial infarct volume <70ml; **and**
age< 80, NIHSS>10, infarct volume <31 ml; **or**	A ratio of volume of ischemic tissue to initial infarct volume <1.8; **and**
Age <80, NIHSS>20, infarct volume< 51 ml	A volume of penumbra >15ml*
Infarct volume was assessed with diffusion-weighted MRI or perfusion CT with automated software(RAPID).	Infarct volume and penumbra regions were assessed from CT perfusion or MRI diffusion and perfusion scans was calculated with automated software (RAPID).
Stroke onset between 6 and 24 hours	Stroke onset between 6 and 16 hours
Prestroke mRS 0 or 1	Prestroke mRS 0-2
No hemorrhagic on CT or MRI	No hemorrhagic on CT or MRI

NIHSS: National Institute of Health Stroke Scale; mRS: modified Rankin scale; *estimated from the volume of tissue for which there was delayed arrival of an injected tracer agent (time to maximum of the residue function exceeding 6 seconds.

Nogueira RG, Jadhav AP, Haussen DC, Bonafe A, Budzik RF, Bhuva P, et al. Thrombectomy 6 to 24 Hours after Stroke with a Mismatch between Deficit and Infarct. New Engl J Med. 2018 Jan 4;378(1):11-21.

Albers GW, Marks MP, Kemp S, Christensen S, Tsai JP, Ortega-Gutierrez S, et al. Thrombectomy for Stroke at 6 to 16 Hours with Selection by Perfusion Imaging. New Engl J Med. 2018 Feb 22;378(8):708-18.

Supplementary Table II. Unit costs, utility weights, disutilities and time to event distributions applied in the health economics model

mRS	Cost of recur stroke hospital[‡]	Cost of Stroke management[^]	Utility[*]
0	$4025	$1431	1
1	$6602	$1431	0.91
2	$11674	$1814	0.76
3	$11674	$1814	0.65
4	$23426	$14027	0.33
5	$23426	$17943	0

	Values	Reference
Probability of fatal stroke	0.028	Bronnum-Hansen et al. 2001[3]
Cost of thrombectomy procedure[‖]	$29198	Arora et al 2018[4]; NHCDC cost report[5]
Cost of MI hospitalisation	$8944	NHCDC cost report[5]
Cost of management post MI	$4302	Turkstra et al 2013[6]
Disutility of having an acute MI	-0.076	Becerra et al 2015[7]
Disutility of having an recurrent stroke	-0.115	Tirschwell et al 2018[8]

	Base case	Parameters	Sensitivity analysis	Parameters	Reference
Recurrent stroke	Exponential	Lambda 0.039	Gompertz	Male: lambda 1.2341; gamma 0.085<br>Female: lambda 6.1442; Gamma 0.112	Mar et al. 2010[9]; Mohan et al 2011[10]
MI	Exponential	Lambda 0.0084	Exponential	Lambda 0.0108	Dhammon et al 2007[11]; Pana et al 2019[2]

[ǂ]informed by reference[5]; [^]informed by reference[4] and included the costs of rehabilitation and nursing home care; [*]informed by reference[12]

[‖]including the costs of acute hospitalisation and thrombectomy

Supplementary Table III. Background non-cardiovascular mortality rate by age

Age	Non-CVD death
25	0.00031
35	0.00068
45	0.001545
55	0.003318
65	0.008087
75	0.024712
85	0.085399
95	0.212265
105	1

CVD: cardiovascular disease

Reference: Australian Bureau of Statistics, Causes of Death Australia 2017.

Supplementary Table IV. Mortality of myocardial infarction by age

Age	Mortality _MI
18	0.157
30	0.157
35	0.157
40	0.157
45	0.154
50	0.142
55	0.179
60	0.2
65	0.228
70	0.297
75	0.348
80	0.477
85	0.63

MI: myocardial infarction

Reference: Cobiac LJ, Magnus A, Lim S, Barendregt JJ, Carter R, Vos T. Which Interventions Offer Best Value for Money in Primary Prevention of Cardiovascular Disease? PLOS ONE. 2012;7(7):e41842.

Supplementary Table V. Baseline characteristics of the unmatched cohort

	EVT					No EVT				
	**All (N=161)**	**DEFUSE neg not matched (N=2)**	**DEFUSE pos not matched (N=28)**	**DAWN neg not matched (N=18)**	**DAWN pos not matched (N=32)**	**All (N=211)**	**DEFUSE neg not matched (N=10)**	**DEFUSE pos not matched (N=41)**	**DAWN neg not matched (N=44)**	**DAWN pos not matched (N=73)**
Age (mean, SD)	67.6 (14.7)	60.5 (20.2)	66.3 (14.1)	70.3 (12.0)	71.9 (13.7)	69.1 (13.5)	72.5 (13.8)	68.2 (13.0)	65.8 (12.8)	70.6 (13.1)
Gender (male, %)	99 (61.5%)	2 (100%)	16 (57.1%)	12 (66.7%)	19 (61.4%)	126 (59.7%)	3 (30.0%)	25 (61.0%)	23 (52.3%)	44 (60.3%)
Baseline NIHSS	16 (12-20)	n.r*	15 (13-18)	6 (4-8)	16 (12-21)	15 (10-19)	19 (18-22)	16 (12-19)	7 (4-9)	16 (13-18)
Baseline core volume	22 (8-43)	n.r*	26 (13-36)	7 (5-15)	27 (9-39)	22 (7-55)	91 (79-96)	23 (11-37)	8 (0.1-30)	21 (8-47)
Penumbra volume	91 (61-120)	n.r*	102 (72-121)	77 (32-110)	85 (57-110)	70 (30-106)	60 (21-93)	77 (59-107)	31 (5-82)	69 (30-99)
Treatment type										
Both EVT and tPA	82 (50.9%)	1 (50%)	15 (53.6%)	12 (66.7%)	14 (43.8%)	0	0	0	0	0
EVT	79 (48.1%)	1 (50%)	13 (46.4%)	6 (33.3%)	18 (56.2%)	0	0	0	0	0
tPA	0	0	0	0	0	16 (7.6%)	0	2 (4.9%)	0	2 (2.7%)
Time to CTP (mins, mean, SD)	331 (83)	538 (94)	627 (84)	584 (73)	637 (86)	387 (94)	527 (72)	439 (64)	561 (73)	448 (51)
Proportion receiving EVT<6 hours	37 (23.0%)	0 (0%)	3 (10.7%)	2 (11.1%)	7 (21.9%)	-	-	-	-	-
Target mismatch	132 (82.0%)	0 (0)	28 (100%)	14 (77.8%)	31 (96.9%)	143 (67.8%)	0 (0)	41 (100%)	18 (40.9%)	59 (80.8%)

Core volume>70ml	20 (12.4%)	1 (50%)	0 (0)	2 (11.1%)	0 (0)	32 (15.2%)	8 (80.0%)	0 (0)	10 (22.7%)	0 (0)
Occlusion site										
ICA	65 (40.4%)	1 (50%)	11 (39.3%)	7 (38.9%)	8 (25.0%)	74 (35.1%)	5 (50.0%)	14 (34.1%)	18 (40.9%)	17 (23.3%)
M1	96 (59.6%)	1 (50%)	17 (60.7%)	11 (61.1%)	24 (75.0%)	137 (64.9%)	5 (50.0%)	27 (65.9%)	26 (59.1%)	56 (76.7%)

n.r.: not reportable; *baseline NIHSS>12; baseline core volume was 92 and 0 ml respectively; penumbra volume was 264 and 0 ml respectively.

Supplementary Table VI. Results from the model by 3-month mRS status_ DAWN criteria

	EVT with DAWN pos			EVT with DAWN neg			No EVT with DAWN pos			No EVT with DAWN neg		
	Total cost	Total QALY	Average cost/QALY	Total cost	Total QALY	Average cost/QALY	Total cost	Total QALY	Average cost/QALY	Total cost	Total QALY	Average cost/QALY
**mRS 0**	$75,178	14.035	$5,356	$74,521	14.021	$5,315	$56,855	14.055	$4,045	$56,281	14.050	$4,006
**mRS 1**	$75,086	12.825	$5,854	$76,500	12.842	$5,957	$56,245	12.786	$4,399	$57,376	12.807	$4,480
**mRS 2**	$75,783	10.765	$7,040	$74,903	10.672	$7,018	$57,966	10.764	$5,385	$55,250	10.622	$5,202
**mRS 3**	$75,470	9.186	$8,216	$75,721	9.194	$8,236	$57,043	9.183	$6,211	$57,282	9.205	$6,223
**mRS 4**	$75,167	4.659	$16,134	$75,047	4.669	$16,073	$57,235	4.664	$12,270	$57,784	4.663	$12,392
**mRS 5**	$74,146	0.140	$528,834	n.a	n.a	n.a	$55,543	0.140	$396,413	$57,148	0.141	$404,498
**mRS 6**	$29,198			$29,198	0		$10,890	0		$10,890	0	

Note: the long-term cost by mRS status was not significantly different across the treatment and perfusion imaging selection criteria. However, the difference in the average cost and QALY was determined by the number of simulated patients within each mRS status.

n.a.: not available since for patients received EVT while being DAWN negative, none of patients achieved the mRS 3 at 3-month from the INSPIRE.

Supplementary Table VII. Results from the model by 3-month mRS status_ DEFUSE 3 criteria

	EVT with DEFUSE 3 pos			EVT with DEFUSE 3 neg			No EVT with DEFUSE 3 pos			No EVT with DEFUSE 3 neg		
	Total cost	Total QALY	Average cost/QALY	Total cost	Total QALY	Average cost/QALY	Total cost	Total QALY	Average cost/QALY	Total cost	Total QALY	Average cost/QALY
**mRS 0**	$74,963	14.046	$5,337	$74,302	14.012	$5,303	$56,268	14.034	$4,009	$56,807	14.042	$4,046
**mRS 1**	$75,184	12.800	$5,874	$75,754	12.791	$5,923	$56,877	12.793	$4,446	$57,200	12.859	$4,448
**mRS 2**	$76,012	10.775	$7,055	$75,055	10.726	$6,998	$57,873	10.786	$5,366	$57,501	10.732	$5,358
**mRS 3**	$75,269	9.179	$8,201	$75,591	9.190	$8,225	$57,085	9.172	$6,224	$56,714	9.179	$6,179
**mRS 4**	$74,549	4.664	$15,985	$75,596	4.668	$16,196	$56,944	4.656	$12,230	$57,705	4.665	$12,370
**mRS 5**	$74,024	0.141	$526,608	$75,579	0.140	$538,609	$56,305	0.142	$397,054	$55,918	0.141	$396,081
**mRS 6**	$29,198	0	n.a	$29,198	0.000		$10,890	0.000		$10,890	0.000	

Note: the long-term cost by mRS status was not significantly different across the treatment and perfusion imaging selection criteria. However, the difference in the average cost and QALY was determined by the number of simulated patients within each mRS status.

Supplementary Table VIII Results of cost-effectiveness analysis_ five-year results

	Thrombectomy procedure		Medical treatment		Thrombectomy procedure		Medical treatment	
	**DEFUSE 3positive**	**DEFUSE 3 negative**	**DEFUSE 3 positive**	**DEFUSE 3 negative**	**DAWN positive**	**DAWN negative**	**DAWN positive**	**DAWN negative**
Total QALYs	3.35	2.30	2.68	2.89	2.88	2.13	2.67	1.76
Total LYs	4.86	3.50	4.60	4.41	4.30	3.21	4.27	3.82
Total costs	$40872	$37601	$21947	$21482	$39534	$36906	$21152	$20083
**Average number of events***								
Deaths	0.207	0.430	0.249	0.284	0.299	0.476	0.304	0.377
MI	0.063	0.045	0.059	0.057	0.056	0.042	0.055	0.050
Stroke	0.284	0.205	0.269	0.258	0.252	0.187	0.250	0.225
Cost of hospitalisation	$32398	$31492	$13919	$13781	$32024	$31298	$13698	$13403
Cost of management	$8474	$6110	$8028	$7701	$7510	$5607	$7454	$6680

*this is the average number of event per patient (not all patients experienced the CVD event).

Supplementary Table IX Results of cost-effectiveness analysis_ Gompertz distribution for probability of recurrent stroke

	Thrombectomy procedure		Medical treatment		Thrombectomy procedure		Medical treatment	
	**DEFUSE 3 positive**	**DEFUSE 3 negative**	**DEFUSE 3 positive**	**DEFUSE 3 negative**	**DAWN positive**	**DAWN negative**	**DAWN positive**	**DAWN negative**
Total QALYs	10.23	7.01	8.20	8.84	8.78	6.49	8.16	5.40
Total LYs	14.70	10.58	13.92	13.35	13.01	9.72	12.92	11.57
Total costs	$53068	$46354	$33467	$32558	$50312	$44955	$31849	$29653
**Average number of events***								
Deaths	0.484	0.628	0.511	0.531	0.543	0.658	0.546	0.594
MI	0.280	0.198	0.263	0.252	0.246	0.183	0.244	0.218
Stroke	0.006	0.004	0.006	0.006	0.006	0.004	0.006	0.005
Cost of hospitalisation	$31065	$30516	$12640	$12572	$30837	$30415	$12516	$12342
Cost of management	$22003	$15838	$20827	$19987	$19475	$14540	$19332	$17311

QALYs: quality-adjusted life years; LY: life years
*this is the average number of event per patient (not all patients experienced the CVD event).

Supplementary Table X. List of published studies examined the cost-effectiveness of late window EVT in large vessel occlusion stroke

Peultier A-C, Pandya A, Sharma R, Severens JL, Redekop WK (2020) Cost-effectiveness of Mechanical Thrombectomy More Than 6 Hours After Symptom Onset Among Patients With Acute Ischemic Stroke. JAMA Network Open 3, e2012476-e2012476.
Peultier A-C, Redekop WK, Allen M, Peters J, Eker OF, Severens JL (2019) Exploring the Cost-Effectiveness of Mechanical Thrombectomy Beyond 6 Hours Following Advanced Imaging in the United Kingdom. *Stroke* **50**, 3220-3227.
Pizzo E, Dumba M, Lobotesis K. Cost-utility analysis of mechanical thrombectomy between 6 and 24 hours in acute ischemic stroke. *Int J Stroke. 2020 Jan; 15(1):75-84.*

References

1. Touze E, Varenne O, Chatellier G, Peyrard S, Rothwell PM, Mas JL. Risk of myocardial infarction and vascular death after transient ischemic attack and ischemic stroke: a systematic review and meta-analysis. Stroke 2005;36:2748-2755.
2. Pana TA, Wood AD, Mamas MA, et al. Myocardial infarction after acute ischaemic stroke: Incidence, mortality and risk factors. Acta Neurol Scand 2019;140:219-228.
3. Bronnum-Hansen H, Davidsen M, Thorvaldsen P, Danish MSG. Long-term survival and causes of death after stroke. Stroke 2001;32:2131-2136.
4. Arora N, Makino K, Tilden D, Lobotesis K, Mitchell P, Gillespie J. Cost-effectiveness of mechanical thrombectomy for acute ischemic stroke: an Australian payer perspective. J Med Econ 2018;21:799-809.
5. Independent Hospital Pricing Authority Australia. National Hospital Cost Data Collection Cost Report: Round 20 Financial Year 2015-16. Available from: https://www.ihpa.gov.au/sites/default/files/publications/nhcdc_cost_report_round_20_financial_year_2015-16_0.pdf. 2018.
6. Turkstra E, Hawkes AL, Oldenburg B, Scuffham PA. Cost-effectiveness of a coronary heart disease secondary prevention program in patients with myocardial infarction: results from a randomised controlled trial (ProActive Heart). BMC Cardiovasc Disord 2013;13:33.
7. Becerra V, Gracia A, Desai K, et al. Cost-effectiveness and public health benefit of secondary cardiovascular disease prevention from improved adherence using a polypill in the UK. Bmj Open 2015;5:e007111.
8. Tirschwell DL, Turner M, Thaler D, et al. Cost-effectiveness of percutaneous patent foramen ovale closure as secondary stroke prevention. J Med Econ 2018;21:656-665.
9. Mar J, Arrospide A, Comas M. Budget impact analysis of thrombolysis for stroke in Spain: a discrete event simulation model. Value Health 2010;13:69-76.
10. Mohan KM, Wolfe CD, Rudd AG, Heuschmann PU, Kolominsky-Rabas PL, Grieve AP. Risk and cumulative risk of stroke recurrence: a systematic review and meta-analysis. Stroke 2011;42:1489-1494.

11. Dhamoon MS, Tai W, Boden-Albala B, et al. Risk of myocardial infarction or vascular death after first ischemic stroke: the Northern Manhattan Study. Stroke 2007;38:1752-1758.
12. Chaisinanunkul N, Adeoye O, Lewis RJ, et al. Adopting a Patient-Centered Approach to Primary Outcome Analysis of Acute Stroke Trials Using a Utility-Weighted Modified Rankin Scale. Stroke 2015;46:2238-2243.
